# *OsEL2* Regulates Rice Cold Tolerance by MAPK Signaling Pathway and Ethylene Signaling Pathway

**DOI:** 10.3390/ijms26041633

**Published:** 2025-02-14

**Authors:** Jiacheng Wu, Xufeng Cao, Xingzhuo Sun, Yulin Chen, Peng Zhang, Yanting Li, Chuan Ma, Lingli Wu, Xin Liang, Qiuping Fu, Lihua Li, Jianqing Zhu, Xiaomei Jia, Xiaoying Ye, Jun Zhu, Rongjun Chen

**Affiliations:** 1 State Key Laboratory of Crop Gene Exploration and Utilization in Southwest China, Rice Research Institute of Sichuan Agricultural University, Chengdu 611130, China; jiachengwu99@163.com (J.W.); chenrj913@163.com (X.C.); sxz15154123375@163.com (X.S.); cchyulin@163.com (Y.C.); zp112611@163.com (P.Z.); 13568771182@163.com (Y.L.); 18981662375@163.com (C.M.); m136276578301@163.com (L.W.); 15608188272@163.com (X.L.); d2229822870@163.com (Q.F.); lilihua1976@tom.com (L.L.); zhujun987@126.com (J.Z.); 2 Demonstration Base for International Science & Technology Cooperation of Sichuan Province, Sichuan Agricultural University, Chengdu 611130, China; zhujianqing@163.com (J.Z.); jiaxiaomei@sicau.edu.cn (X.J.); 70166@sicau.edu.cn (X.Y.); 3 Crop Ecophysiology and Cultivation Key Laboratory of Sichuan Province, Rice Research Institute of Sichuan Agricultural University, Chengdu 611130, China

**Keywords:** *OsEL2*, cold, MAPK, ethylene, Rboh, tryptophan metabolism, phenylalanine metabolism, monoterpene synthesis

## Abstract

Low temperature stress represents a significant abiotic stress factor affecting rice yields. While the structure and some of the functions of cell cycle protein-dependent protein kinase inhibitor (CKI) family proteins have been the subject of study, their relevance to cold tolerance in rice has been less investigated. In this study, we cloned *OsEL2* (*LOC_Os03g01740*) and constructed anti-expression lines of this gene. The resulting lines exhibited significant cold sensitivity and displayed greater oxidative damage than wild type Nippobare (Nip). However, the activities of antioxidant enzymes, such as catalase (CAT), were significantly elevated in *OsEL2*-AX plants in comparison to Nip following exposure to 4 °C stress. RNA sequencing revealed the presence of 18,822 differential genes, with the majority of them being expressed with temporal specificity. The Kyoto Encyclopedia of Genes and Genomes (KEGG) pathway enrichment analysis revealed that a considerable number of differentially expressed genes (DEGs) are involved in the metabolism of amino acids, lipids, and terpenoids. Weighted gene co-expression network analysis (WGCNA) revealed a close relationship between the genes in the turquoise and light green modules and rice cold tolerance traits. These genes were predominantly enriched in terpene metabolism and the metabolism of various plant secondary metabolites, suggesting that *OsEL2* influences rice cold tolerance through the metabolism of these two classes of substances. An analysis of the genes within these two modules using transcription factor (TF) enrichment and KEGG enrichment revealed that they are predominantly regulated by mitogen-activated protein kinase (MAPK) and ethylene signaling pathways. Furthermore, we found that tryptophan metabolism, phenylalanine metabolism, and monoterpene synthesis were enriched in down-regulated pathway enrichment analysis. In addition, we also found that the MAPK signaling pathway was enriched in the KEGG enrichment analysis of AX2 with Nip. The results demonstrate that anti-expression of *OsEL2* is associated with a notable decline in rice tolerance to cold stress.

## 1. Background

Rice, as one of the world’s major food crops, provides food rations for more than half of the earth’s population [1,2]. As a species originating in the tropics and subtropics, rice is a cereal crop sensitive to low-temperature stress [3]. It is more susceptible to low-temperature stress at higher latitudes. Low temperature stress, as one of the major factors limiting the distribution and yield of rice, can be categorised into two types: cold (0–15 °C) and freezing (<0 °C) [4]. With the growing population, the demand for rice yields is also increasing. The rising demand for rice yields has contributed to the expansion of rice cultivation, and the probability of suffering from low-temperature stress in the process of expanding to higher latitudes is also increasing, increasing the likelihood of a reduction in rice yields [5].

Low temperatures have a detrimental impact on rice, leading to a range of adverse effects, including a low germination rate, plant death, and a reduction in spike fertility. Low temperatures have been shown to exert a significant negative impact on crop yields, particularly during the reproductive growth stage. This environmental stress can lead to pollen sterility and poor ovule development, resulting in a reduced seed setting rate and smaller seeds, and ultimately reduced yields [6,7,8]. Studies have demonstrated that cold damage can lead to yield losses ranging from 0.810 to 2.740 tons/ha, resulting in a potential reduction in grain yields of up to 38.6% [9]. The incidence of cold stress has been demonstrated to impact the chlorophyll content of rice, consequently influencing the rate of photosynthesis [10,11]. Additionally, physiological changes occur, such as alterations in enzyme activity, changes in soluble sugar content, and accumulation of reactive oxygen species (ROS). An accumulation of excessive ROS results in oxidative damage to cells, which in turn affects rice metabolism [12]. However, ROS are important signaling molecules that play a significant role in the response of plants to biotic or abiotic stresses [13,14,15]. ROS are involved in almost all processes related to development, stress, and immunity [16,17]. O_2_^−^ is produced by the respiratory burst oxidase homologous protein (Rboh), which catalyses the transfer of electrons from nicotinamide adenine dinucleotide phosphate (NADPH) to O_2_ [18]. Subsequently, O_2_^−^ is catalysed by superoxide dismutase to form H_2_O_2_ [19]. The C-terminus of plant Rboh is identical to gp91^phox^, whereas the N-terminal structure contains calcium-binding EF hand motifs, which are more similar to the human RanGTPase-activating protein [20,21]. In contrast to the mammalian gp91^phox^, the plant Rboh is also active in the absence of additional cytoplasmic components and is directly stimulated by Ca²⁺ [22]. To date, Rboh has been identified in a number of plant species, including rice, maize, chili peppers, and citrus fruits [23,24,25,26,27,28].

Over a long period of evolution, plants have developed a number of survival strategies to adapt to low temperature stress. Metabolites play a significant role in regulating cold tolerance in plants. The unsaturation of plant cell membranes is increased at low temperatures in order to ensure the normal functioning of the plasma membrane. Arachidonic acid (AA) is transferred from triglycerides (TAG) to polar lipids when the ambient temperature drops rapidly [29], which suggests that AA plays an important role in rapidly increasing the degree of unsaturation in the plasma membrane. The lack of AA in chloroplast membranes results in an increase in membrane rigidity, which ultimately leads to increased chloroplast damage in cold stress [30]. Phenylalanine metabolism has been shown to be actively involved in plant resistance to cold stress [31]. Liu et al. [32] found that differentially expressed genes were enriched to the monoterpene metabolic pathway between the treated and control groups after cold stress.

The MAPK pathway is a highly conserved regulatory pathway in eukaryotes that plays a pivotal role in regulating cellular activities. Furthermore, it is a key factor in the cold tolerance of rice [33,34,35]. The MAPK pathway comprises three sequential components, each involved in phosphorylation and subsequent activation: MAPK kinase kinase (MEKK or MKKK), MAPK kinase (MEK or MKK), and MAPK [35]. It has been reported that in Arabidopsis, the AtCRLK1-AtMEKK1-AtMKK2-AtMPK4/6 pathway positively regulates freezing tolerance [36]. Furthermore, the MKKK proteins AtANP1 and AtMPK3 have been shown to initiate a phosphorylation cascade under cold stress [37].

In eukaryotes, the cell cycle is tightly regulated by Cyclins (Cyc) and their corresponding cell cycle protein-dependent protein kinases (CDKs). When CKIs bind to CDKs, or Cyc, or Cyc-CDK complexes, they alter the cell cycle by inhibiting their activity [38,39]. Plant CKIs can be classified into two groups, namely, KIP-related proteins (KRPs) and SIAMESE-related proteins (SMRs) [40]. The KIP-related proteins have been identified and analysed in a variety of plants, including rice, maize, and Arabidopsis [41,42]. Overexpression of *KRP1* leads to dwarfing of *Arabidopsis thaliana*, reducing the cell number and increasing the cell size [43]. As indicated by prior research, seven KRP family members have been identified in rice, and they have been designated *KRP1* to *KRP7* [44,45,46]. Overexpression of *KRP1* inhibits cell proliferation and grain filling by suppressing cell endorepublication [44]. *KPR3* was highly expressed in cellularised endosperm two days after fertilisation, suggesting that this gene plays an important role in the development of endosperm [47]. Overexpression of *OsiICK6* led to a substantial decrease in plant growth, pollen viability, and fruit set, as well as the occurrence of abaxial leaf curling [46].

Despite a number of studies exploring the function of CKIs, few studies have addressed their roles in stress responses. The findings of this study indicated that the anti-expression of *OsEL2* resulted in a significant reduction in cold tolerance in rice.

## 2. Results

### 2.1. Bioinformatics Analysis of OsEL2

**Figure 1:**
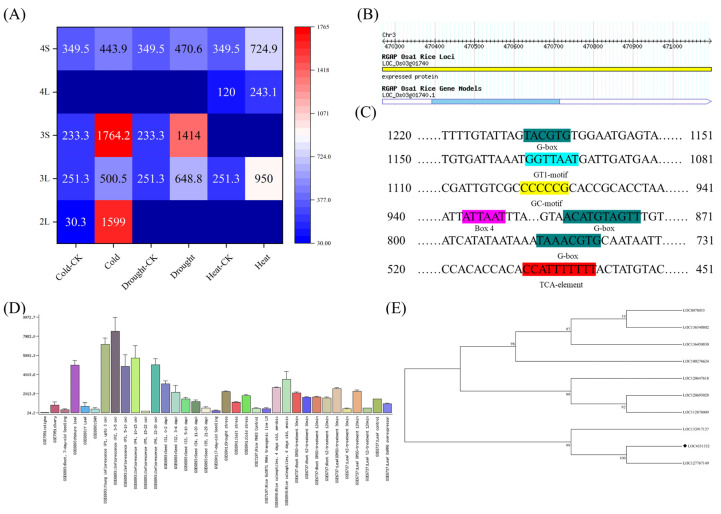
Bioinformatics analysis of *OsEL2*. (**A**) Expression of *OsEL2* in different tissue sites in different developmental periods. The numbers in the squares represent the expression of the genes. Squares without a number indicate that the corresponding sample was not detected. (**B**) The information of *OsEL2*. (**C**) Analysis of elements associated with the promoter region of *OsEL2*. The same colors represent the same cis-acting elements, the names of the elements are indicated below the color block. (**D**) Expression pattern of *OsEL2* in RiceGE. (**E**) Phylogenetic tree of *OsEL2*. The ◆ labelled in the figure represents *OsEL2*.

We analysed gene expression in different tissue sites in different developmental periods under different abiotic stresses using a gene microarray and found that *OsEL2* (*LOC_Os03g01740*) was significantly up-regulated in leaves at the seedling stage and in spikelets at the booting period after low temperature treatment (Figure 1A). Recent studies have demonstrated that *OsEL2*, a novel cell cycle regulatory gene, is associated with the reported SIAMESE (SIM) gene in *Arabidopsis thaliana* [41]. To ascertain whether this gene plays a role in regulating stress responses in rice, further studies were conducted. *OsEL2* is located on rice chromosome 3, is intronless, has an open reading frame size of 324 bp, and encodes a peptide chain containing 107 amino acid residues (Figure 1B). Its promoter contains not only basic cis-acting elements, but also cis-acting elements related to abiotic stress response, such as G box, GT1-motif, GC-motif, Box 4, and TCA-element (Figure 1C). This implies that *OsEL2* may be responsive to abiotic stresses.

The expression of the *OsEL2* gene in rice organs at different times and under different stress treatments was investigated using the RiceGE (Gene Expression Graphics, http://signal.salk.edu/cgi-bin/RiceGE, accessed on 28 September 2024) online database, as illustrated in Figure 1D. The findings revealed that *OsEL2* was expressed in multiple plant tissues, including the ovaries, leaves, inflorescences, and seeds. Moreover, its expression was markedly elevated under conditions of drought, salt, and low temperature. Based on the above results, we hypothesised that *OsEL2* plays an important role in the regulation of cold tolerance in rice.

To explore the homology of this gene across species, homologous sequences of *OsEL2* were identified in different species through the use of NCBI’s discontigous megablast sequence comparison method, and a total of 34 sequences were retrieved. A total of 10 sequences with higher query cover, which were all EL2 or EL2-like genes and SMR-like genes, were selected to construct a phylogenetic tree using MEGA11 (11.0.13). As illustrated in Figure 1E, *OsEL2* exhibits high homology to the cell cycle protein-dependent kinase inhibitor analog (*LOC127767149*) of *Oryza glaberrima* Steud. Even the closest homologous gene, *LOC127767149*, has a step length of 100, implying a large difference between *OsEL2* and homologous genes in other species. Based on the above findings, we chose this gene for further study.

### 2.2. The OsEL2 Gene Is Localised Within the Cell Nucleus

**Figure 2:**
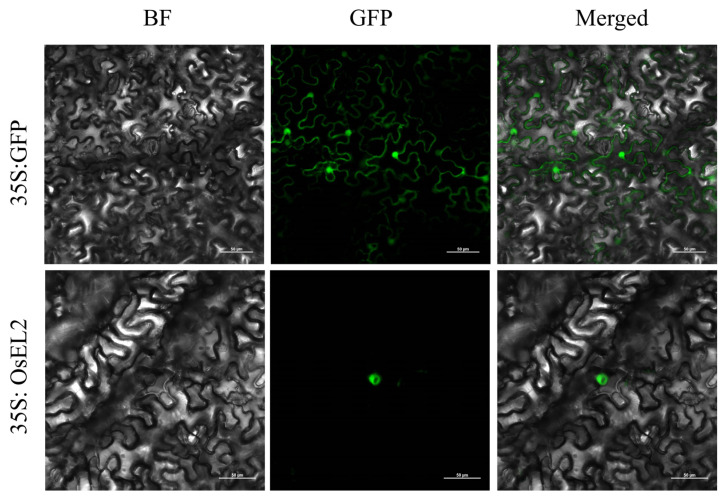
Subcellular localisation of OsEL2 in *Nicotiana benthamiana*. The location of the green fluorescence represents the position of the corresponding recombinant protein in the cell.

To explore the localisation of OsEL2 in cells, we constructed an OsEL2-GFP expression vector. The expression vector was transiently expressed in *Nicotiana benthamiana* by Agrobacterium, and it was observed that OsEL2 was localised in the nucleus (Figure 2).

### 2.3. OsEL2 Responds to Multiple Abiotic Stresses

**Figure 3:**
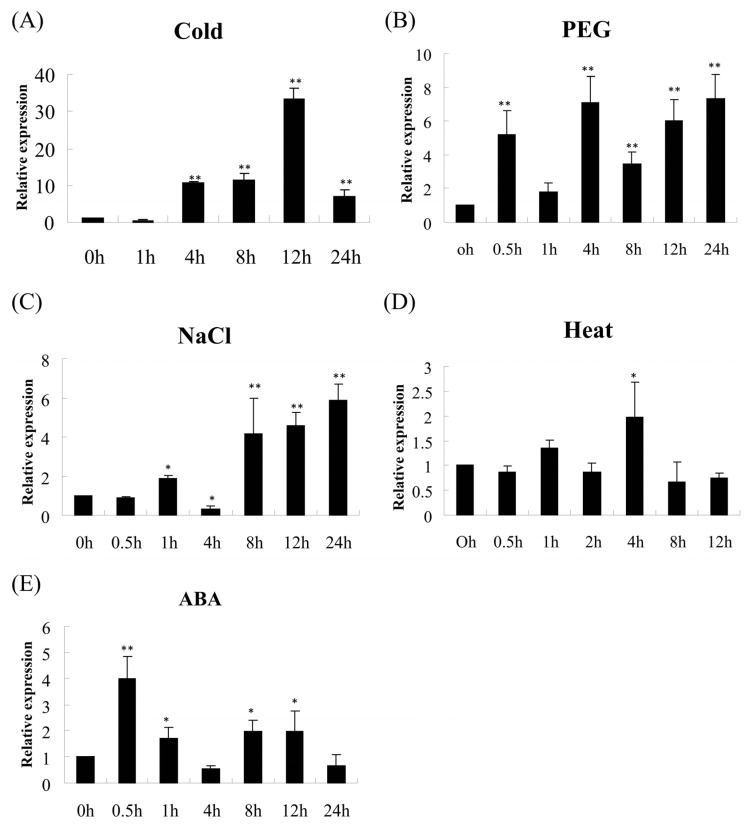
The relative transcript level of *OsEL2* in Nip at the three-leaf stage under abiotic stresses. (**A**) 4 °C. (**B**) 20% (*w*/*v*) PEG6000. (**C**) 150 mM NaCl. (**D**) 45 °C. (**E**) 30 μM ABA. *n* = 3. * *p* < 0.05, ** *p* < 0.01.

To gain further insight into the response of *OsEL2* to different abiotic stresses, we subjected Nip plants at the three-leaf stage to a range of abiotic stresses and examined the relative transcript levels of *OsEL2*. As illustrated in Figure 3, *OsEL2* demonstrated a response to various abovementioned stresses, including cold, heat, salt, and drought stresses, as well as ABA treatment. *OsEL2* transcript levels exhibited a significant increase after 1 h of cold stress and remained at a higher level after 1 h of the remaining time of cold treatments. A significant up-regulation of *OsEL2* was observed at 0.5 h after PEG treatment, subsequently declining to a level that was not significantly different from that at 0 h. Thereafter, *OsEL2* expression increased and remained at a higher level. Furthermore, *OsEL2* was significantly up-regulated at 0.5 h after ABA treatment, and its expression level was not significantly changed at 4 h and 24 h after ABA treatment. The rest of the time points exhibited higher transcript levels compared to 0 h. The transcript level of *OsEL2* was found to be significantly up-regulated under cold stress conditions. This result was consistent with the findings above, as well as with the results of previous studies [41].

### 2.4. Anti-Expression of OsEL2 Reduces Cold Stress Tolerance in Rice

**Figure 4:**
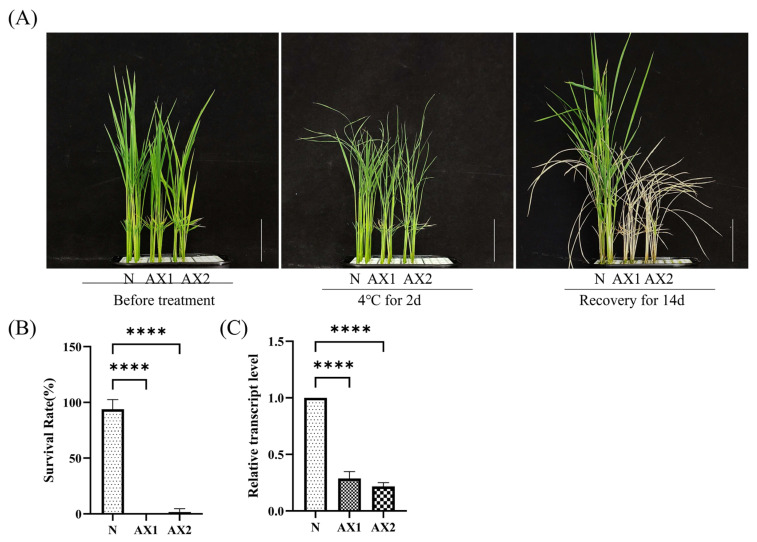
Detection of cold tolerance in *OsEL2*-AX rice. (**A**) Phenotype of 2-week-old Nip and transgenic seedings before and after 4 °C treatments for 2 d. *n* = 3. Each experiment contained 16 biological replicates per line (**B**) Survival rate of seedings after 4 °C treatment. *n* = 3. (**C**) Identification of *OsEL2* anti-expression lines. *n* = 3. Asterisks indicate significant differences according to the one-way ANOVA test, **** *p* < 0.0001. Scale bar = 5 cm.

Based on the above results, we speculated that this gene was associated with cold stress tolerance in rice and therefore constructed anti-expression lines of *OsEL2* for further investigation. To investigate the role of *OsEL2* in cold tolerance in rice, we constructed a vector containing the 35S promoter and the full length of the CDS of *OsEL2*, thus obtaining anti-expression lines of *OsEL2*. To select anti-expressing lines for subsequent experiments, we selected lines that were significantly lower than Nip by examining the relative transcript levels of *OsEL2* in each line (Figure 4C). Finally, we selected two anti-expressing lines (AX1, AX2).

In order to examine the response of the *OsEL2* anti-expression strain (*OsEL2*-AX) to cold stress, we cultivated Nip and two anti-expression lines (AX1, AX2) under standard conditions. Subsequently, we transferred them to 4 °C for a period of two days and then returned the seedlings to normal conditions for a further 14 days. The results demonstrated that *OsEL2*-AX exhibited pronounced chlorosis during the cold treatment phase, displaying grey-green leaves that withered and died within the initial two days of the recovery period (Figure 4A,B). These observations indicated that *OsEL2*-AX displayed clear cold-sensitive traits.

### 2.5. Identification of Differentially Expressed Genes (DEGs) Between Nip and Anti-Expression of OsEL2 Under Cold Stress

**Figure 5:**
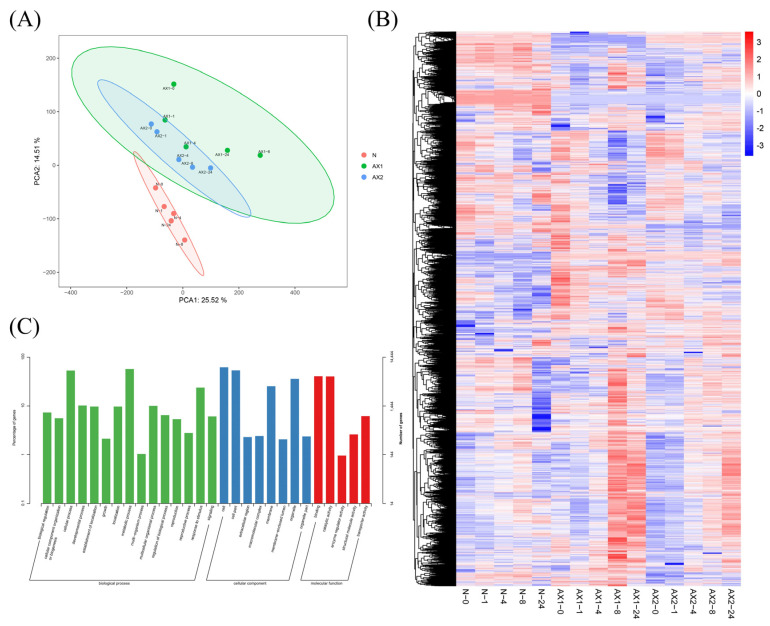
Identification and analysis of DEGs. (**A**) PCA analysis of all DEGs. (**B**) Heatmap of all DEGs. (**C**) GO enrichment analysis of all DEGs.

In order to gain a deeper understanding of the molecular mechanism by which *OsEL2* regulates cold tolerance in plants, we performed a transcriptome analysis utilising RNA sequencing technology. As illustrated in Figure 5A of the principal component analysis, a notable distinction was observed between *OsEL2*-AX and Nip, whereas a considerable degree of resemblance was observed between the two anti-expression lines. At the same time, we also analysed the identification of DEGs between Nip and the two anti-expression lines. A total of 18,822 DEGs were screened in the three lines treated at 4 °C for 0, 1, 4, 8, and 24 h (Figure 5B, Supplementary Material S1). Gene ontology (GO) enrichment analysis revealed that a considerable number of DEGs were enriched in cellular components and biological processes. The most enriched biological processes were metabolic and cellular processes, indicating that the anti-expression of *OsEL2* significantly affected cellular components and the level of generated metabolites (Figure 5C).

### 2.6. Anti-Expression of OsEL2 Affects Multiple Metabolic Pathways

**Figure 6:**
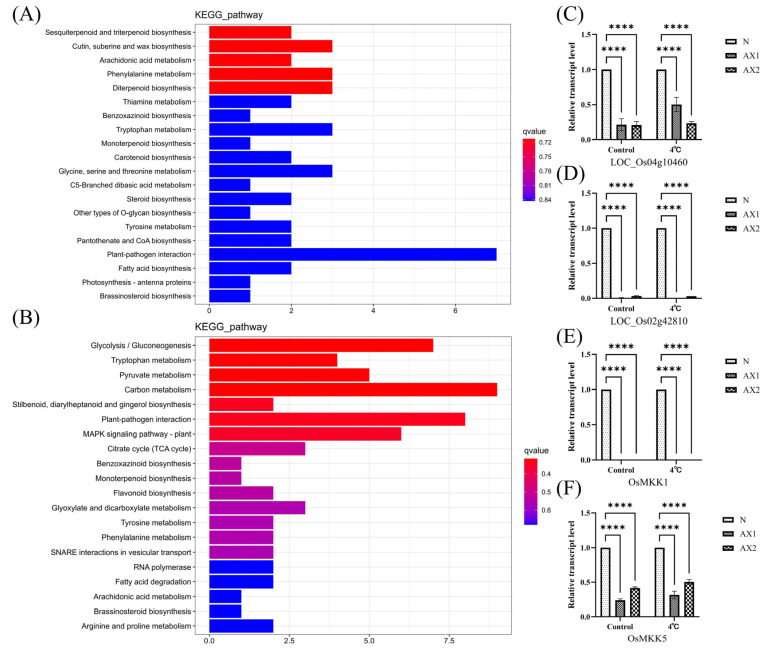
KEGG enrichment analysis of down-regulated pathways. (**A**,**B**) Down-regulated pathway enrichment analysis. (**A**) AX1 vs. Nip. (**B**) AX2 vs. Nip. (**C**–**F**) Relative transcript levels of genes associated with down-regulated pathways, *n* = 3. (**C**) *LOC_Os04g10460*. (**D**) *OsPLIM2a* (*LOC_Os02g42810*). (**E**) *OsMKK1* (*LOC_Os06g05520*). (**F**) *OsMKK5* (*LOC_Os06g09180*). **** *p* < 0.0001.

KEGG enrichment analysis showed that a large number of metabolism-related pathways were enriched in the down-regulated pathways, among which tryptophan metabolism, phenylalanine metabolism, and monoterpene synthesis were enriched in the KEGG enrichment analyses of the two anti-expression lines with Nip, with a high q-value (Figure 6A,B). *LOC_Os04g10460* was found to be enriched in pathways associated with tryptophan and phenylalanine metabolism. *OsPLIM2a* (*LOC_Os02g42810*) was enriched in pathway associated with monoterpene synthesis. Furthermore, as previously stated in Section 2.8, the transcript levels of genes encoding key enzymes involved in these metabolic pathways were markedly reduced under cold stress conditions in comparison to Nip, providing additional evidence for the downregulation of these pathways. In addition, we found an enrichment of MAPK signaling pathway in the KEGG down-regulation pathway enrichment analysis of AX2 vs. Nip, with a high q-value. The expression levels of *OsMKK1* and *OsMKK5* were significantly reduced in both the AX1 vs. Nip and AX2 vs. Nip comparisons. Therefore, we selected genes in these pathways that showed significant differences in both anti-expression lines compared to Nip for RT-qPCR (Figure 6C–F). The results were consistent with those of the transcriptome data, and the transcript levels of the target genes in the anti-expression lines were significantly lower than that of Nip; we hypothesised that anti-expression of *OsEL2* may modulate cold tolerance in rice through these pathways.

### 2.7. MAPK Signaling Pathway and Ethylene Signaling Pathway Regulate Cold Tolerance

**Figure 7:**
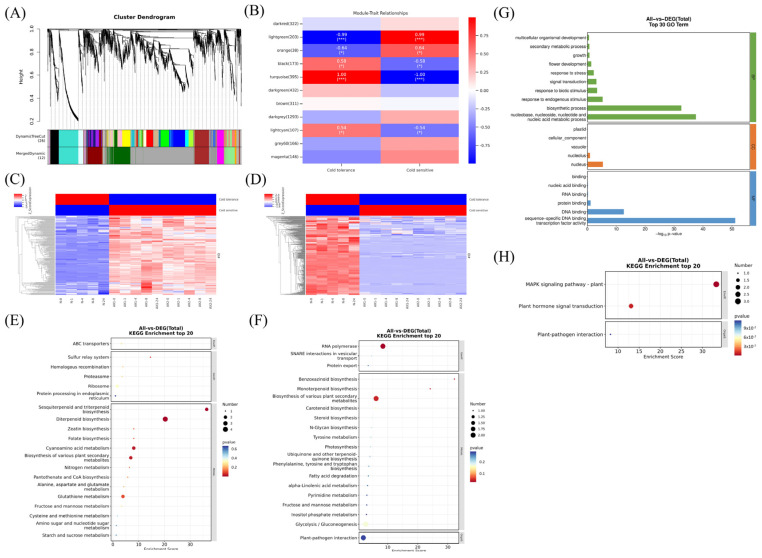
MAPK signaling pathway and ethylene signaling pathway regulate cold tolerance. (**A**) Clustering trees for DEGs. Different colors represent different gene modules. (**B**) Modular trait association analysis. (**C**) Cluster analysis of light green modules. (**D**) Cluster analysis of turquoise modules. (**E**) KEGG enrichment analysis of light green modules. (**F**) KEGG enrichment analysis of turquoise modules. (**G**) GO enrichment analysis of TF. (**H**) KEGG enrichment analysis of TF. * *p* < 0.05, *** *p* < 0.001.

In order to investigate the key genes and their upstream regulatory networks that have a significant impact on cold tolerance traits, a weighted gene co-expression network analysis (WGCNA) was first performed on the transcriptome data. We filtered the genes based on low expression fluctuations (σ ≤ 0.75), which resulted in the identification of 3820 DEGs. To satisfy the power law distribution of the degree distribution of the gene co-expression network, we selected 30 as the power value. The correlation coefficient R² = 0.78 for power = 30 satisfies the construction of a scale-free network (Supplementary Material S2, Figure S1A,B). Consequently, we proceeded to the subsequent stage of the analysis. As illustrated in Figure 7A and Supplementary Figure S1C, 3820 DEGs were categorised into various modules. Through the analysis of module–module correlations, the final classification of DEGs into 12 modules was determined (Supplementary Material S3). The grey modules represent a set of genes that could not be attributed to any module and, thus, lacked any meaningful reference. The turquoise and light green modules exhibited robust negative and positive correlations with cold-sensitive traits, respectively, with high statistical significance. Furthermore, the transcript levels of the genes within these modules were diametrically opposed to those of Nip (Figure 7B–D and Supplementary Material S2 Figure S1D–K), suggesting a close relationship between these two modules and cold tolerance in rice. Interestingly, we found that *OsEL2* was not categorised into these two modules, but rather into the brown module, a module that did not show a strong correlation with the trait, suggesting that this gene affects cold tolerance in rice by influencing the expression of other genes. We subsequently performed a KEGG enrichment analysis of the genes within these two modules, which revealed that the genes within light green were predominantly involved in sesquiterpenoid and triterpenoid biosynthesis, diterpenoid biosynthesis, cyanoamino acid metabolism, and biosynthesis of various plant secondary metabolites (Figure 7E). The genes in turquoise, in contrast, were primarily involved in RNA polymerase, benzoxazinoid biosynthesis, monoterpenoid biosynthesis, and biosynthesis of various plant secondary metabolites (Figure 7F). These results suggest that *OsEL2* affects rice cold tolerance mainly by influencing the metabolism of rice secondary metabolites, including the metabolism of terpenoids. We subsequently performed a TF enrichment analysis of the genes within these two modules, which resulted in the identification of 44 enriched transcription factors (Supplementary Material S4). GO enrichment analysis revealed that these transcription factors are mainly involved in biological processes such as biosynthesis processes; nucleobase, nucleoside, nucleotide, and nucleic acid metabolic processes; DNA binding; and sequence-specific DNA binding transcription factor activity (Figure 7G). KEGG enrichment analysis showed that TF was mainly enriched in the MAPK signaling pathway and plant hormone signal transduction, and the two transcription factors enriched in plant hormone signal transduction were both ethylene signal transcriptional regulators. The above results indicate that the MAPK signaling pathway and ethylene signaling pathway are closely related to *OsEL2* in regulating cold tolerance in rice (Figure 7H).

### 2.8. The Anti-Expression of OsEL2 in Seedlings Subjected to Cold Stress Results in Severe ROS Damage Despite the Elevated Antioxidant Capacity

**Figure 8:**
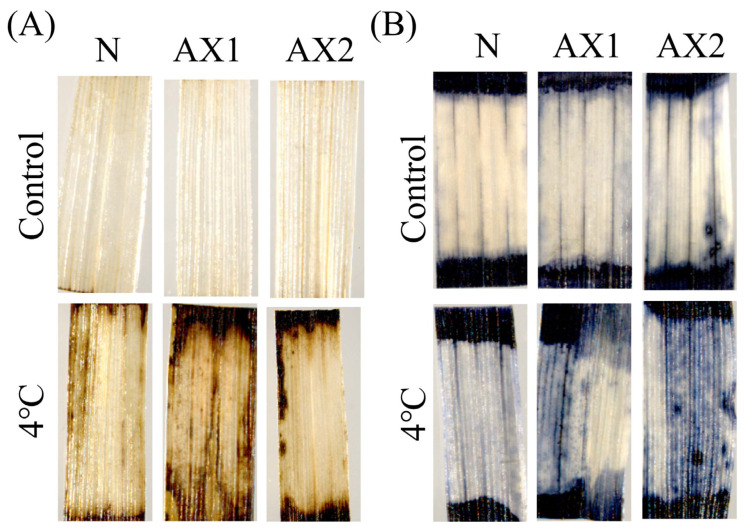
ROS accumulation in Nip and *OsEL2*-AX transgenic plants. (**A**) DAB staining. (**B**) NBT staining. *n* = 3.

Low temperatures result in the accumulation of ROS in plants. The antioxidant capacity of transgenic rice was determined by testing different levels of indicators. Initially, the accumulation of ROS in rice seedlings before and after a 4 °C treatment was examined using DAB and NBT staining. As illustrated in Figure 8A,B, a large number of stained spots indicated that the levels of O_2_^−^ and H_2_O_2_ accumulation in *OsEL2*-AX were significantly higher than those in Nip following a 4 °C treatment. Therefore, *OsEL2*-AX exhibited greater ROS damage after cold stress treatment.

**Figure 9:**
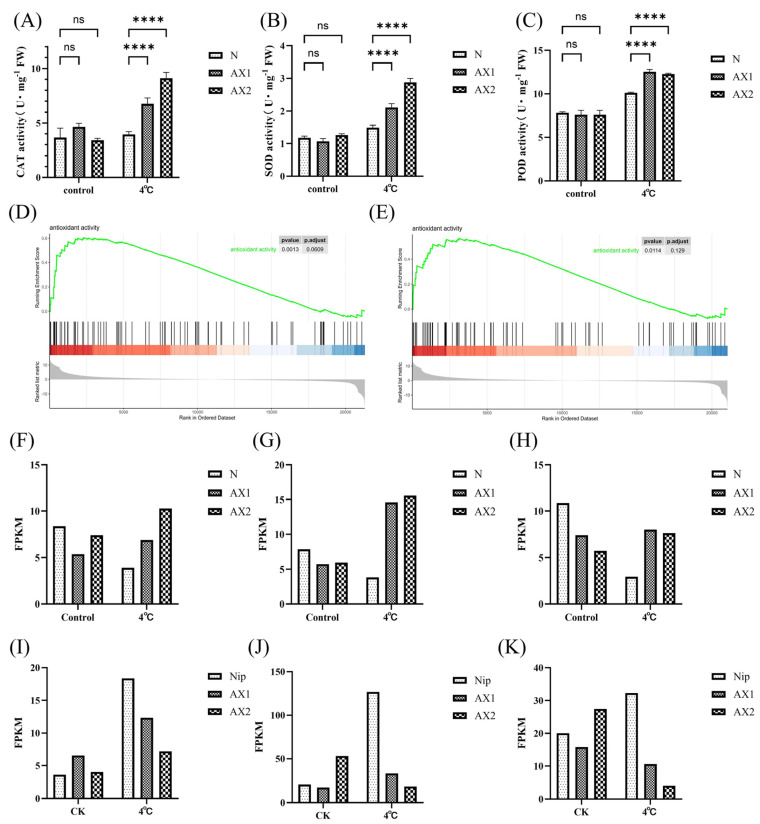
Determination of physiological parameters before and after 4 °C for 1 d. (**A**) CAT activity. *n* = 3. (**B**) SOD activity. *n* = 3. (**C**) POD activity. *n* = 3. (**D**,**E**) GSEA analysis of the antioxidant capacity. The color transition from blue to red is indicative of low to high gene expression. (**D**) AX1 vs Nip. (**E**) AX2 vs Nip. (**F**–**H**) FPKM of selected Rboh. (**F**) *Osrboh A* (*LOC_Os01g53294*). (**G**) *Osrboh B* (*LOC_Os01g25820*). (**H**) *Osrboh G* (*LOC_Os09g26660*). (**I**) *OsOASA2* (LOC_Os03g15780). (**J**) *OsPAL6* (LOC_Os04g43800). (**K**) *OsTPS10* (LOC_Os03g22634). Asterisks indicate significant differences between transgenic lines and Nip using a two-way ANOVA test, **** *p* < 0.0001. ns stands for no significant difference.

It is noteworthy that the examination of catalase (CAT), peroxidase (POD), and superoxide dismutase (SOD) enzyme activities following cold stress treatment revealed a significant elevation in all three enzymes (Figure 9A–C). This finding aligns with the elevated antioxidant capacity of the *OsEL2* anti-expression strain, as compared to Nip, which was identified by the results of transcriptome analysis using gene set enrichment analysis (GSEA) (Figure 9D–E). It has been demonstrated [48] that ROS levels in the leaves of Rboh knockdown tomato plants decreased significantly in line with a reduction in Rboh enzyme activity. Subsequently, we screened for Rboh enzymes and examined and analysed their relative expression in the RNA-seq results in detail. The results revealed that the relative expression of these genes was markedly elevated in both AX1 and AX2 lines following treatment at 4 °C compared to Nip (Figure 9F–H). In addition to enzymatic reactions, non-enzymatic substances (such as isoflavones, tryptophan, monoterpenes, and so forth) can also scavenge ROS [49,50,51]. The transcript levels of genes encoding key enzymes involved in the metabolic pathway for the synthesis of these substances were found to remain unaltered or even to undergo a significant reduction following cold treatment (Figure 9I–K). Accordingly, we postulated that the elevation in CAT, SOD, and POD enzyme activities and the pronounced ROS damage observed in the anti-expression lines of *OsEL2* could be attributed to the marked up-regulation of Rboh enzyme activities and reductions in these metabolic pathways, which resulted in an excessive accumulation of ROS.

### 2.9. Anti-Expression of OsEL2 Affects Agronomic Traits in Rice

**Figure 10:**
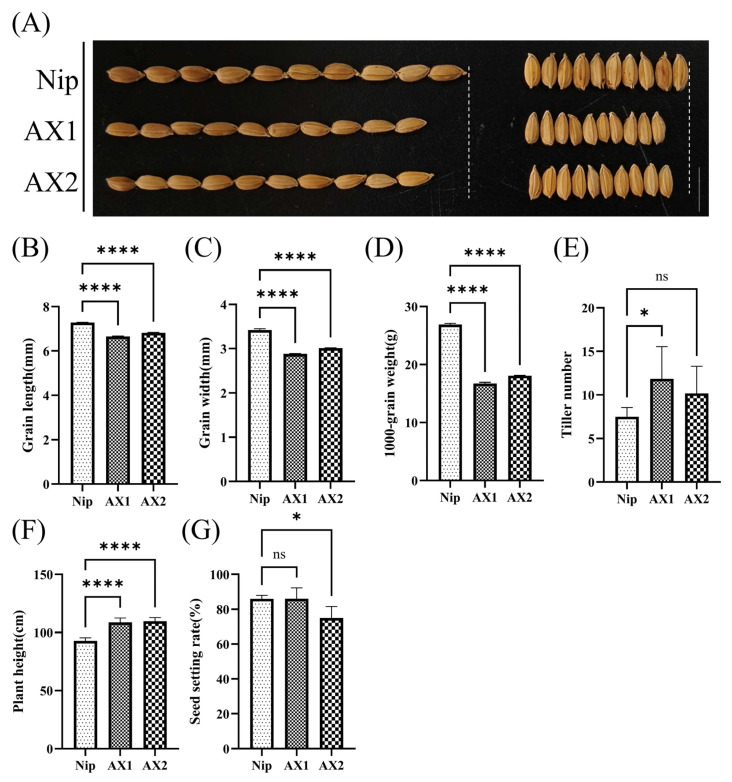
Determination of agronomic traits. (**A**) Rice seed phenotypes of Nip and AX lines. *n* = 3. Scale bar = 1 cm. (**B**–**G**) Statistics of agronomic traits of Nip and AX lines including the grain length, grain width, 1000-grain weight, tiller number, plant height, and seed setting rate. (**B**) Grain length. *n* = 3. (**C**) Grain width. *n* = 3. (**D**) 1000-grain weight. *n* = 3. (**E**) Tiller number. *n* = 4. (**F**) Plant height. *n* = 4. (**G**) Seed setting rate. *n* = 4. Asterisks indicate significant differences between transgenic lines and Nip using a two-way ANOVA test, * *p* < 0.05, **** *p* < 0.0001. ns stands for no significant difference.

In this study, we found that *OsEL2* significantly affected the agronomic characteristics of rice. As shown in Figure 10A–D, anti-expression of *OsEL2* significantly reduced the grain size of rice and significantly decreased the 1000-grain weight. In addition, anti-expression of *OsEL2* increased the number of tillers and plant height in rice, with the number of tillers in AX1 being significantly more than that in Nip, and the plant height in both AX1 and AX2 being significantly higher than that in Nip (Figure 10E,F). Anti-expression of *OsEL2* also reduced the seed setting rate of the anti-expression lines, with AX2 having a significantly lower seed setting rate than Nip.

## 3. Discussion

Due to its immobility, rice has developed a sophisticated network of adaptive responses to a variety of natural stresses, enabling it to survive and reproduce. The cell cycle, as an important part of plant growth and development, also plays an important role in the plant’s response to external environmental stimuli [52,53]. The cell cycle is regulated by CDKs and their corresponding cell cycle protein partners [52,53]. CKIs can inhibit the activity of CDK/CYC complex proteins by binding to them, ultimately affecting the cell cycle [54,55]. However, there are few studies related to the response of CKI proteins to cold stress. In this study, the *OsEL2* gene of CKIs in rice could significantly affect the tolerance of rice to low temperature.

The content of ROS, as one of the important products of plant responses to abiotic stress, is closely related to stress tolerance. Excessive ROS can cause severe damage to plants, but low concentrations of ROS play an important role as signaling molecules in plants [56]. Therefore, plants need to ensure that their ROS levels are in an appropriate range. When plants are under abiotic stress, antioxidant enzymes are transcriptionally activated to maintain ROS at an appropriate level. CAT, SOD, and POD all play important roles in scavenging ROS [57,58,59,60]. In our results, both DAB and NBT staining showed that *OsEL2*-AX accumulated more ROS after cold stress, even though the enzyme activities of CAT, SOD, and POD increased. The results of the RNA sequencing demonstrated a notable elevation in the relative expression of specific Rboh in comparison to Nip following exposure to cold stress. This observation led to the formulation of the hypothesis that the elevated levels of multiple Rboh enzymatic activities may be a contributing factor to the observed outcomes.

The role of various metabolites in plant resistance to low-temperature stress is equally important. Melatonin, a tryptophan derivative, has been demonstrated to enhance the cold tolerance of melons by increasing their antioxidant capacity and photosynthetic efficiency [61]. An et al. [62] demonstrated that the anti-expression of *OsJRL* led to the upregulation of the phenylalanine metabolism pathway in rice, which subsequently enhanced the plant’s tolerance to cold temperatures. Liu et al. [32] discovered that DEGs from chrysanthemums subjected to low-temperature stress and maintained under standard conditions were enriched in monoterpene synthesis through KEGG enrichment analysis. Concurrently, the metabolites of these three metabolic pathways also participate in the scavenging of ROS [49,50,51]. In this study, tryptophan metabolism, phenylalanine metabolism, and monoterpene synthesis were enriched in down-regulated pathways. We hypothesised that the anti-expression of *OsEL2* may affect cold tolerance in rice through these metabolic pathways.

The MAPK signaling pathway, as one of the important signaling pathways in plants, plays an important role in response to abiotic stresses. Zhang et al. demonstrated that the MAPK cascade, ABA, and Ca^2+^ signaling can collectively regulate cold tolerance in azalea plants [63]. Xie et al. found that *OsMPK3* and *OsMPK6* are activated at low temperatures of 12 °C to regulate cold tolerance in rice [64]. We found that the relative transcript levels of *OsMKK1* and *OsMKK5* were significantly lower than those of Nip in two anti-expressing lines of *OsEL2*, both before and after cold treatment. We hypothesised that *OsEL2* may affect rice cold tolerance through these two genes. In addition, we identified three MAPK signaling pathway-related transcription factors among the transcription factors obtained from the enrichment, further demonstrating that this signaling pathway plays an important role in rice cold tolerance.

Ethylene plays a key role in plant growth and development and is also involved in regulating plant responses to abiotic stresses [65]. It has been shown that ethylene plays an important role in plant responses to cold stress and that ethylene levels are negatively correlated with plant cold tolerance [66,67,68]. In this study, we identified two ethylene signaling transcriptional regulators involved in the regulation of genes strongly associated with regulatory traits, suggesting that the ethylene signaling pathway plays an important role in *OsEL2* regulation of cold tolerance in rice.

The present study focused on the transcriptional level to investigate the mechanism by which *OsEL2* regulates cold tolerance in rice, and did not explore its metabolism in any depth.

## 4. Methods

### 4.1. Plant Materials, Growth Conditions, and Abiotic Stress Treatments

The plant material *Oryza sativa* L. subsp. japonica cv. Nipponbare (Nip) was used in this experiment as the wild-type rice. *OsEL2*-AX transgenic rice plants were generated in the Nip background. Screened with hygromycin B and PCR detection, T10 generation plants were used in this experiment [69]. All methods were performed in accordance with the relevant guidelines and regulations.

Rice was grown in a nutrient solution and placed in a constant temperature greenhouse at 26 °C under cyclic conditions of 16 h of light and 8 h of darkness. After 2 weeks of cultivation, 16 consistent seedlings for each rice line were selected and subjected to stress treatments, namely, cold treatment (4 °C) for 2 days. After the stress treatment, the seedlings were transferred to a non-stress environment. Then, 14 days later, the growth status of the seedlings was observed. Seedlings that were still green and growing were considered to be surviving.

### 4.2. Analysis of the OsEL2 Gene

The *OsEL2* gene information was obtained from the Rice Genome Annotation Project (RGAP, http://rice.uga.edu/, accessed on 6 July 2024) and the National Center for Biotechnology Information (NCBI, http://www.ncbi.nlm.nih.gov/, accessed on 28 September 2024) database. The expression of the *OsEL2* gene in rice organs at disparate times and under varied stress treatments was investigated using the RiceGE (Gene Expression Atlas) online database. The homologous sequences of *OsEL2* were identified in different species through the use of the NCBI’s discontigous megablast sequence comparison method, and a total of 34 sequences were retrieved. Then, 10 sequences with higher query cover were selected for phylogenetic tree construction using MEGA11 (11.0.13).

### 4.3. Protein Subcellular Localisation

*OsEL2* was cloned using the primers *OsEL2*-GFP-F and *OsEL2*-GFP-R. The *OsEL2*-GFP vector was transformed into *Agrobacterium tumefaciens* EHA105. Transformed Agrobacterium strains were activated and injected into the lower epidermis of *Nicotiana benthamiana* leaves and subsequently cultured under low light for 2 d. The 35S:GFP was used as a control. GFP-derived fluorescence was analysed using confocal laser scanning microscopy (Nikon A1-90i, LSCM, Tokyo, Japan). The excitation/emission wavelengths were as follows: GFP (488 nm/505 to 575 nm).

### 4.4. Plasmid Construction and Rice Transformation

To generate the *OsEL2*-AX lines, the entire *OsEL2* coding region was connected to the vector pCAMBIA1300 (a vector with some modifications; primers: *OsEL2*-AX-F and *OsEL2*-AX-R). These constructs were introduced into Nip via the Agrobacterium-mediated transformation method [70].

### 4.5. Isolation of DNA and RNA and RT-qPCR

Fresh leaves were sampled at different time periods (0 h, 0.5 h, 1 h, 2 h, 4 h, 8 h, 16 h, 24 h) and immediately frozen in liquid nitrogen. Genomic DNA was extracted from rice seedlings via the CTAB method [71]. The total RNA was extracted using Trizol reagent (Invitrogen, Burlington, ON, Canada) according to the manufacturer’s instructions. The reverse transcription was conducted using a PrimeScript™ RT Reagent kit with a gDNA Eraser kit (+GDNA wiperVazyme, Beijing, China), and the cDNA was stored at −20 °C. The relative expression levels of target genes were determined based on the 2^−ΔΔCt^ method, and the Ubiquitin gene of rice (*LOC_Os01g22490*) was used as an internal control [72]. All primers are listed in Supplementary Material S2 Table S1. The relative transcript levels of *OsEL2* in anti-expression lines were quantified using the primers F-*OsEL2*-F and F-*OsEL2*-R.

### 4.6. Measurement of the Physiological Parameters

The two-week-old seedlings were used for cold treatment. After 24 h of the treatments, the physiological and biochemical indicator parameters of plants were determined. CAT enzyme activity was measured with a CAT Activity Assay Kit (Produced by Solarbio^®^ LIFE SCIENCE, Beijing, China). SOD and POD enzyme activity were measured with corresponding enzyme activity kits (Produced by Grace Biotechnology, Suzhou, China). Leaves were placed in 1 mg/mL DAB and 6 mM NBT staining solution and incubated at 28 °C for 10 h in light [73]. Anhydrous ethanol was used to remove chlorophyll. The accumulation of hydrogen peroxide and superoxide anion O^2−^ was observed under a stereo microscope (Carl Zeiss AG, Oberkochen, Germany).

### 4.7. RNA Extraction and RNA-Seq

RNA high-throughput sequencing and subsequent data analysis were performed by TsingkeBiotechnology Co., Ltd. (Wuhan, China). The Nip, AX1, AX2 lines were selected for sequencing at five discrete time points. Samples were taken at 0, 1, 4, 8, and 24 h after cold treatment, with six seedlings sampled at each time point. The raw image data files obtained from high-throughput sequencing (Illumina Novaseq X Plus, Illumina, Inc., San Diego, CA, USA) were analysed by CASAVA Base Calling and converted into raw data. Transcriptome QC results are shown in Supplementary Material S2 Tables S2 and S3. The designated genome was used as a reference for sequence comparison and subsequent analysis in this study. The reference genome can be accessed at the following URL: https://riceome.hzau.edu.cn/dev/download/IRGSPMSU.gff (accessed on 19 August 2024). Sequence alignment of clean reads with the reference genome was conducted using Hisat2 (v2.2.1) [74] to obtain positional information on the reference genome or gene, as well as sequence feature information specific to the sequenced sample. Transcripts were assembled and FPKM was calculated using StringTie software (v2.0.4) [75] with the objective of predicting expression levels. Differential expression analysis was conducted between sample sets using DEseq2 (v1.26.0) [76] in order to obtain the set of differentially expressed genes under the two biological conditions. The screening criteria employed were a fold change of ≥2 and an FDR (false discovery rate) of <0.01. Furthermore, hierarchical cluster analysis was performed on the screened differentially expressed genes in order to cluster genes with the same or similar expression patterns. The PCA was conducted using the FactoMineR package (v2.11) in R (v3.6.5) [77]. GSEA analysis was enriched by gsea [78] and ranked by the log2 ratio of classes method, with FDR and FWER (family-wise error rate) values taken as 1.00. Gene ontology (GO) enrichment analysis of the differentially expressed genes (DEGs) was implemented by the GOseq R packages (v3.20) based on Wallenius non-central hyper-geometric distribution [79], which adjusts for gene length bias in DEGs. KOBAS (v2.3.4) [80] software was used to test the statistical enrichment of differential expression genes in KEGG [81] pathways.

### 4.8. WGCNA Analysis and Related Enrichment Analysis

The analysis of the transcriptome sequencing results was conducted using the WGCNA tool in OEcloud (https://cloud.oebiotech.com, accessed on 27 December 2024) [82,83], with the standard deviation threshold set to 0.75, the cut sensitivity set to 2, and the weighting parameters, the module merge threshold, and the minimum number of genes in a module set to their adaptive values. The module characteristic genes and traits were analysed using Pearson’s correlation algorithm to calculate the correlation coefficients and *p* values. The modules related to each trait were then identified based on the thresholds of absolute value of correlation coefficients greater than or equal to 0.3 and a *p* value less than 0.05. For each trait-related module, the correlation between the module gene expression and the corresponding trait (gene significance, GS) was calculated. The correlation between the module gene expression and the module characteristic gene (eigengene) was also calculated, and a scatter plot was drawn based on the above two values. TF enrichment analysis was performed by PlantTFDB (https://planttfdb.gao-lab.org/, accessed on 27 December 2024) [84]. GO and KEGG enrichment analyses of modular feature genes and TFs were performed using the OEcloud tools at https://cloud.oebiotech.com, accessed on 27 December 2024.

### 4.9. Measurement of Agronomic Traits

Four rice plants of each line in the field were selected for measuring plant height and the number of tillers. The main spikelets of these rice plants were collected to determine the seed setting rate. For each line, 500 grains were randomly collected and analysed for grain length and width using an automatic rice seed analyser (JLM, mini1600, Shanghai, China), and the weight was measured using an electronic balance (NJNONITALAB, DNA233B, Nanjing, China). The procedure was repeated three times [85].

## 5. Conclusions

In this study, we demonstrated that anti-expressing *OsEL2* lines exhibited sensitivity to cold stress. Subsequent transcriptome analysis revealed that the gene influenced rice cold tolerance by modulating the MAPK and ethylene signaling pathways, which in turn affected the metabolism of various secondary metabolites and terpenes. Tryptophan metabolism, phenylalanine metabolism, and monoterpene synthesis pathways were found to be impacted, leading to altered ROS clearance. The findings of this study suggest a potential mechanism at the transcriptional level through which *OsEL2* affects cold tolerance in rice.

## Data Availability

All data are illustrated with graphs and Supplementary Materials. All plant experiments were performed at our affiliated institutions. The data sets used and/or analysed in this study are available on request from the corresponding author.

## Supplementary Materials

The following supporting information can be downloaded at: https://www.mdpi.com/article/10.3390/ijms26041633/s1.
