# Supplementary material for: OsEL2 Regulates Rice Cold Tolerance by MAPK Signaling Pathway and Ethylene Signaling Pathway
Source: Int J Mol Sci. 2025 Feb 14;26(4):1633. doi: 10.3390/ijms26041633 (PMC11855578; doi:10.3390/ijms26041633)
Supplement: Supplementary file 1 [file ijms-26-01633-s001.zip › Supplement S2.pdf]

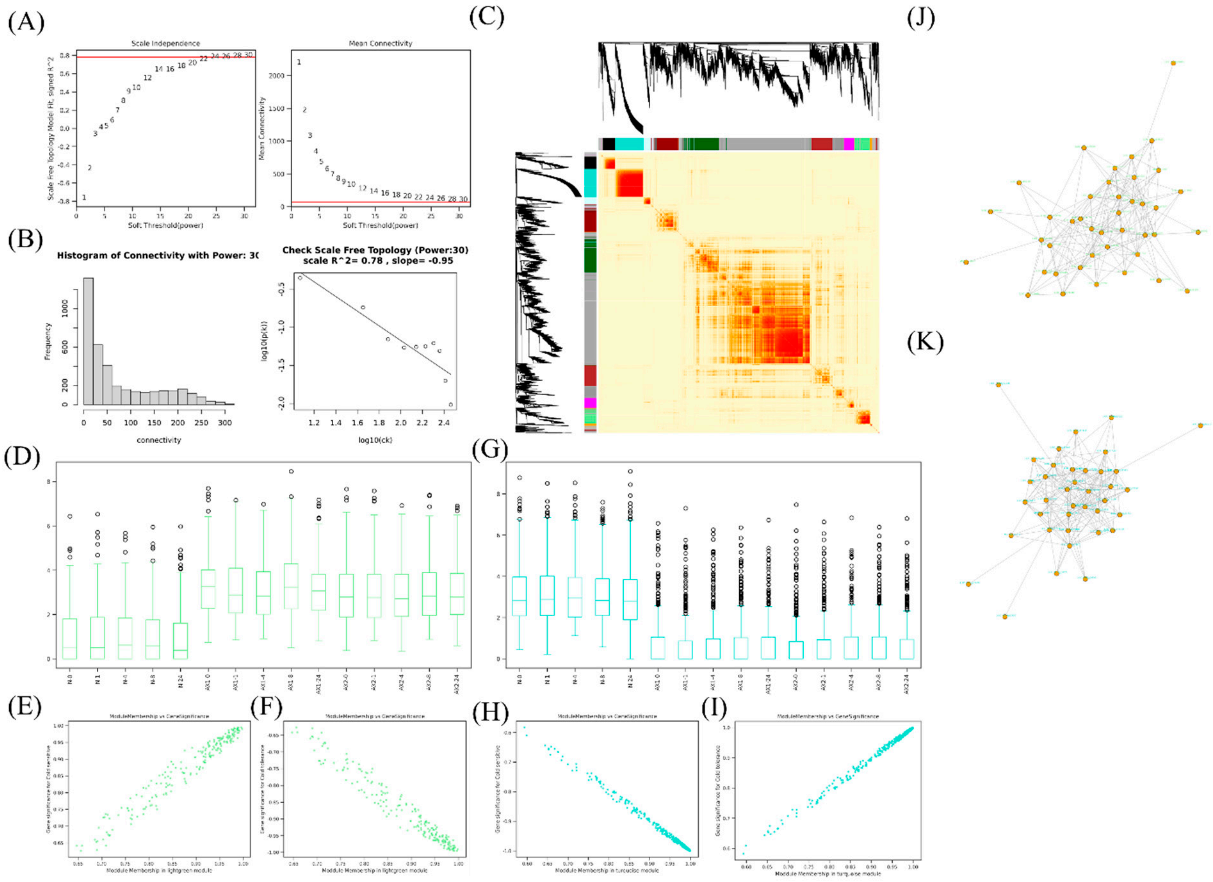

Supplementary Figure S1 Screening and analysis of genes related to cold tolerance. (A) Screening of weighting parameters. (B) Network connectivity distribution and power law distribution validation. (C) Tomplot of DEGs. (D-F) Expression of DEGs of lightgreen module in different lines and their association with cold tolerance. (G-I) Expression of DEGs of turquoise module in different lines and their association with cold tolerance. (J) The core gene network of the lightgreen module. (K) The core gene network of the turquoise module.

Supplementary Table S1 PCRprimer

Primer Name	Sequences
OsEL2-AX-F	gtaccgaattcccggggatccATGTCCGCCTCGCCCGAG
OsEL2-AX-R	tggagaggacagcccaagcttTCAGGTATGGGTGGGCTTGTT
OsEL2-GFP-F	tggagaggacagcccaagcttATGTCCGCCTCGCCCGAG
OsEL2-GFP-R	ctcaccatgaccggtggatccGTATGGGTGGGCTTGTTGGTT
F-OsEL2-F	GAAGCTCCTCTTCGACCCAG
F-OsEL2-R	TTTGGAGGTGAAGGTTGGCA
OsMKK1-F	TGGCGCCAGAAAGAATCAGT
OsMKK1-R	CACCAGTGGCCAATTCTAGC
OsMKK5-F	GAATCTTGGCAAGCAGGGTG
OsMKK5-R	GAAGGCAGTAGCCGACGAAG
UBQ-RTQ-F	ACCACTTCGACCGCCACTACT
UBQ-RTQ-R	ACGCCTAAGCCTGCTGGTT
LOC_Os04g10460-F	ACAAGACACTGTTGGGCCAA
LOC_Os04g10460-R	CAGTCGCTTCAGCATCAAGC
LOC_Os02g42810-F	GGGACCAGGAAGCAACAACT
LOC_Os02g42810-R	CTCGAGCCCAAGTCCTTTGT

Note: Lowercase letters of the primers in the table are the homology arm sequences of the expression vectors.

Supplementary Table S2 Sequencing Data Statistics

Samples	Clean reads	Clean bases	GC Content	%≥Q30
AX1-0	30,684,243	9,150,864,173	53.34%	96.98%
AX1-1	28,299,722	8,410,877,616	53.90%	96.92%
AX1-24	32,590,777	9,675,123,483	52.66%	97.04%
AX1-4	32,316,955	9,581,983,311	53.50%	97.07%
AX1-8	28,827,505	8,560,157,147	52.76%	96.92%
AX2-0	31,739,908	9,429,009,446	53.82%	97.07%
AX2-1	26,879,890	7,939,775,996	53.21%	96.95%
AX2-24	30,816,813	9,114,458,167	52.62%	97.00%
AX2-4	30,477,863	8,964,422,855	53.33%	96.67%
AX2-8	31,277,038	9,240,633,284	52.54%	97.01%
N-0	29,060,127	8,606,283,034	53.31%	97.00%
N-1	27,509,981	8,143,706,173	53.56%	96.90%
N-24	26,838,603	7,991,872,270	52.31%	96.99%
N-4	26,969,491	8,010,709,440	52.11%	97.05%
N-8	29,292,998	8,743,016,549	53.14%	96.83%

Supplementary Table S3 comparison efficiency statistics of RNA-Seq

ID	Total Reads	Mapped Reads	Uniq Mapped Reads	Multiple Map Reads	Reads Map to '+'	Reads Map to '-'
AX1-0	61,368,486	59,351,459 (96.71%)	56,723,547 (92.43%)	2,627,912 (4.28%)	29,399,737 (47.91%)	29,406,112 (47.92%)
AX1-1	56,599,444	54,927,964 (97.05%)	52,797,541 (93.28%)	2,130,423 (3.76%)	27,245,356 (48.14%)	27,255,101 (48.15%)
AX1-24	65,181,554	63,144,480 (96.87%)	59,551,345 (91.36%)	3,593,135 (5.51%)	31,267,623 (47.97%)	31,256,497 (47.95%)
AX1-4	64,633,910	62,676,257 (96.97%)	59,639,484 (92.27%)	3,036,773 (4.70%)	31,033,536 (48.01%)	31,032,999 (48.01%)
AX1-8	57,655,010	55,585,076 (96.41%)	53,646,554 (93.05%)	1,938,522 (3.36%)	27,657,674 (47.97%)	27,653,988 (47.96%)
AX2-0	63,479,816	61,889,443 (97.49%)	59,025,937 (92.98%)	2,863,506 (4.51%)	30,652,984 (48.29%)	30,658,757 (48.30%)
AX2-1	53,759,780	52,404,009 (97.48%)	49,074,282 (91.28%)	3,329,727 (6.19%)	25,830,748 (48.05%)	25,829,598 (48.05%)
AX2-24	61,633,626	59,889,289 (97.17%)	56,181,586 (91.15%)	3,707,703 (6.02%)	29,567,797 (47.97%)	29,557,611 (47.96%)
AX2-4	60,955,726	59,201,884 (97.12%)	56,768,326 (93.13%)	2,433,558 (3.99%)	29,371,791 (48.19%)	29,374,338 (48.19%)
AX2-8	62,554,076	60,728,786 (97.08%)	57,496,612 (91.92%)	3,232,174 (5.17%)	29,987,453 (47.94%)	29,983,534 (47.93%)
N-0	58,120,254	57,401,998 (98.76%)	53,949,702 (92.82%)	3,452,296 (5.94%)	28,328,446 (48.74%)	28,328,297 (48.74%)
N-1	55,019,962	54,356,279 (98.79%)	52,513,155 (95.44%)	1,843,124 (3.35%)	27,016,502 (49.10%)	27,024,603 (49.12%)
N-24	53,677,206	52,910,379 (98.57%)	49,469,742 (92.16%)	3,440,637 (6.41%)	26,056,891 (48.54%)	26,051,494 (48.53%)
N-4	53,938,982	53,266,720 (98.75%)	51,066,207 (94.67%)	2,200,513 (4.08%)	26,402,473 (48.95%)	26,406,363 (48.96%)
N-8	58,585,996	56,615,459 (96.64%)	54,126,184 (92.39%)	2,489,275 (4.25%)	28,079,706 (47.93%)	28,081,193 (47.93%)
